# Muscle, Skin and Core Temperature after −110°C Cold Air and 8°C Water Treatment

**DOI:** 10.1371/journal.pone.0048190

**Published:** 2012-11-06

**Authors:** Joseph Thomas Costello, Kevin Culligan, James Selfe, Alan Edward Donnelly

**Affiliations:** 1 Centre for Physical Activity and Health Research, Department of Physical Education and Sport Sciences, University of Limerick, Castletroy, Limerick, Ireland; 2 Institute of Health and Biomedical Innovation, Queensland University of Technology, Kelvin Grove, Queensland, Australia; 3 Department of Surgery, Mid-Western Regional Hospital, Dooradoyle, County Limerick, Ireland; 4 Department of Allied Health Professions, University of Central Lancashire, Preston Lancashire, United Kingdom; Universidad Europea de Madrid, Spain

## Abstract

The aim of this investigation was to elucidate the reductions in muscle, skin and core temperature following exposure to −110°C whole body cryotherapy (WBC), and compare these to 8°C cold water immersion (CWI). Twenty active male subjects were randomly assigned to a 4-min exposure of WBC or CWI. A minimum of 7 days later subjects were exposed to the other treatment. Muscle temperature in the right vastus lateralis (n = 10); thigh skin (average, maximum and minimum) and rectal temperature (n = 10) were recorded before and 60 min after treatment. The greatest reduction (P<0.05) in muscle (mean ± SD; 1 cm: WBC, 1.6±1.2°C; CWI, 2.0±1.0°C; 2 cm: WBC, 1.2±0.7°C; CWI, 1.7±0.9°C; 3 cm: WBC, 1.6±0.6°C; CWI, 1.7±0.5°C) and rectal temperature (WBC, 0.3±0.2°C; CWI, 0.4±0.2°C) were observed 60 min after treatment. The largest reductions in average (WBC, 12.1±1.0°C; CWI, 8.4±0.7°C), minimum (WBC, 13.2±1.4°C; CWI, 8.7±0.7°C) and maximum (WBC, 8.8±2.0°C; CWI, 7.2±1.9°C) skin temperature occurred immediately after both CWI and WBC (P<0.05). Skin temperature was significantly lower (P<0.05) immediately after WBC compared to CWI. The present study demonstrates that a single WBC exposure decreases muscle and core temperature to a similar level of those experienced after CWI. Although both treatments significantly reduced skin temperature, WBC elicited a greater decrease compared to CWI. These data may provide information to clinicians and researchers attempting to optimise WBC and CWI protocols in a clinical or sporting setting.

## Introduction

Whole body cryotherapy (WBC) is a treatment involving very short exposures to extreme cold, and is growing in popularity amongst athletes and coaches [1], [2], [3]. Most WBC protocols repeatedly expose minimally dressed individuals to extremely cold dry air (−110°C to −140°C) in an environmentally controlled room for a short duration of time (2–4 min) [4]. Although the use of cryotherapy or therapeutic tissue cooling is a common form of treatment dating back to ancient Greece [5], WBC is a relatively novel modality of cryotherapy. The first WBC chamber was built in Japan in the late 1970s, but it was only introduced to Europe in 1982 and America in the last decade [6].

A range of claims, based around these thermoregulatory responses, have been made about the benefits of WBC. Currently, the evidence base supporting these claims is extremely limited [4]. Studies examining WBC are traditionally limited, in terms of quality and statistical power, or published in non-English literature. Initial WBC studies have reported a reduction in creatine kinase activity after training [7], [8], total oxidative status in plasma [9] as well as an increase in the anti-inflammatory cytokines IL-10 [10] and IL-6 [9], [10]. Hausswirth and colleagues [2] have recently reported that three WBC sessions accelerated recovery from exercise induced muscle damage (EIMD), but it has also been shown that WBC administered 24 h after eccentric exercise, is ineffective in alleviating muscle soreness or enhancing muscle force recovery [1]. Despite the increasing popularity and use of WBC in sports medicine, randomised controlled studies regarding its efficacy are sparse [4]. Presently, clinicians and sporting organizations are exposing individuals to these extreme temperatures based on anecdotal evidence and very little is known regarding its effectiveness or the physiological changes that occur during or after the treatment [1].

Similarly, despite its widespread adoption in an attempt to alleviate some of the physiologic and functional deficits associated with EIMD and to treat some clinical conditions, the use of cold water immersion (CWI) remains controversial owing in part to the lack of data regarding underlying mechanisms [11], [12]. Although there is much confusion around how much cooling is clinically adequate the basic premise of cryotherapy is to cool injured or damaged tissue [13]. Previous research has demonstrated the mechanisms that underpin CWI such as reductions in blood flow and muscle, skin and core temperature [11] but currently there is a paucity of published research [14], [15] addressing the thermodynamics of WBC. Furthermore, as thermal conductance in water can be as much as three times greater than in air [16], these cooling modalities (WBC and CWI) have a different thermal conduction property and therefore a different skin, muscle and core cooling potential.

In cryotherapy research there is perhaps no more relevant and divisive question than ‘what is the optimal modality, temperature and duration required to elicit the required physiological response?’ For example, in order to reduce nerve conduction velocity, and consequently pain, the magnitude of tissue cooling following cryotherapy is critically important. Nerve conduction velocity has been shown to progressively reduce concomitantly with skin temperature [17]. Similarly, a reduction of 5–15°C in tissue temperature, with the critical level of skin temperature less than 12°C, is required to achieve an analgesic response [13]. Cooling has also been shown to decrease the dynamic contractile force by 4–6% for each 1°C reduction in muscle temperature [18], alter EMG activity during submaximal leg extensions [19] and reduce power output in humans [20]. In addition, if administered incorrectly or for a prolonged period of time, clinicians and researchers need to be aware that cryotherapy can result in cold injury, with cases such as ice burn [21] and even amputation [22] previously being reported within the literature, following crushed ice application and a continuous flow cryotherapy unit respectively. Consequently, despite the optimal tissue temperature following cryotherapy still to be established, it is pivotal that practitioners are familiarised with the thermodynamics of different cooling modalities.

The majority of claims regarding the effectiveness of WBC refer to the ability of the treatment to reduce muscle, skin and core temperature. These claims have yet to be substantiated. To our knowledge, no researchers have sought to simultaneously establish the effects of WBC on muscle, skin and core temperature and compare it to the more commonly used method of cryotherapy, CWI. Therefore, the purpose of this study was to investigate and compare the effects of two modalities of cryotherapy (WBC and CWI) on muscle (vastus lateralis), skin and core (rectal) temperature after an identical duration of treatment. It was hypothesized that CWI would facilitate a greater reduction in all three temperature (muscle, skin and core) measurements, primarily due to the thermal conductivity of water compared to air.

## Materials and Methods

These experiments were conducted according to the Helsinki Declaration (1964: revised in 2001) and the protocol was approved by the local ethics committee (University of Limerick Research Ethics Committee, Limerick, Ireland. Ref. 08/55).

### Subjects

Twenty healthy active males volunteered to participate in this study. Ten subjects completed the intramuscular component of the study (mean ± SD; age = 23.8±2.7 yr, height = 180.9±5.8 cm, mass = 87.2±17.8 kg, body mass index (BMI) = 26.5±4.3 kg/m^2^, mid thigh skinfold = 10.5±5.9 mm, body fat = 23.4±8.7% measured via dual energy x-ray absorptiometry [Lunar iDXA™ GE Healthcare, Chalfont St Giles, Bucks., UK with enCORE™ 2007 v.11 software]). A further ten subjects (mean ± SD; age = 26.5±4.9 yr, height = 183.5±6.0 cm, mass = 90.7±19.9 kg, BMI = 26.8±5.0 kg/m^2^, body fat = 23.0±9.3%) completed the skin and core component of the study. All subjects were moderately trained (exercised a minimum of 3 times per week) and between the age of 18–35. Subjects were familiarized with the experimental procedures and associated risks, completed a medical questionnaire and gave their written informed consent prior to participation. Subjects were excluded if they had any contradiction to cryotherapy including Raynaud’s disease. All subjects were required to refrain from smoking and consumption of alcohol and caffeine 12 hours prior to each laboratory session. In addition, subjects were tested at the same time of day for each trial (separated by 7–10 days), and did not undertake exercise for 24 hours prior to each laboratory session.

### Experimental Protocol

This study was a randomized controlled crossover design. The subjects were assigned, using a random numbers generator, to start with either the WBC or CWI. A minimum of seven days later the subject repeated the other treatment. We have previously described the methodology for the WBC exposure [1]. The WBC exposure was administered in a specially built, temperature-controlled unit (Zimmer Elektromedizin, Germany), which consists of two rooms (−60 and −110°C). The temperature of the therapy room remained at a constant level (−110±3°C [mean ± SD]), and the air in the room was dry and clear. Subjects entered and stood in the first room (−60±3°C) for 20 sec before entering the second room (−110±3°C) for 3 min and 40 sec. Subjects were instructed by the trained machine operator to walk slowly around the chamber and to flex and extend their elbow and fingers throughout the exposure. In the chamber, subjects wore shorts, two pairs of gloves and their nose and mouth were covered with a surgical mask; their ears were covered with a woollen headband and they wore their own dry shoes and socks. All jewellery, piercings, glasses and contact lenses were removed before entry to the chamber.

The temperature and duration of the CWI exposure was similar to other studies in the literature [11], [23] and a protocol similar to our previous work was employed [24]. Following the baseline recordings subjects, wearing only shorts, were seated in a tank filled with cold water (8°C ±0.3°C) and immersed to the level of the sternum for 4 min. A duration of 4 min was chosen as it replicated the duration of WBC exposure. Immediately after CWI, subjects were asked to towel-dry their body, change into dry shorts and transfer to an adjacent laboratory for post tests. The temperature of the water was measured throughout using a digital aquarium thermometer. The ambient temperature of the laboratory during each testing session (WBC and CWI) was 22.0±0.5°C and subjects spent 20 min acclimatising to the room before the commencement of testing.

### Muscle Temperature

The temperature of the vastus lateralis in the right limb was recorded manually on a Medical Precision Thermometer (DM852, Ellab A/S, Hvidovre, Denmark), with an accuracy of 0.1°C. A flexible intramuscular temperature probe (MAC flexible probe, Ellab, Denmark) was inserted through an indwelling flexible cannula (venflon 18GA Becton Dickinson, Sweden) into the muscle in the direction of the muscle fibres and advanced 0.5 cm beyond the end of the cannula into the muscle [25]. Subjects thigh skinfold (Harpenden Skinfold Caliper Baty International, West Sussex, UK) was measured, prior to insertion, and the probe was inserted 3 cm below the subcutaneous fat layer (skinfold x 0.5) [26] by a trained physician. The location of the probe in the vastus lateralis was verified during pilot tests by the use of a GE Logiq e ultrasound scanner ultrasound (GE Medical, Wauwatosa, WI, USA). Muscle temperature data (3 cm subcutaneous) was recorded pre, post treatment and every 10 min thereafter. During data collection subjects remained in a semi reclined position for 60 min before after both treatments. Baseline data was recorded at the end of this 60 min period. A 60 min was chosen as pilot tests demonstrated this to be the maximum time a subject could comfortably sit in a semi reclined position with an indwelling muscle probe inserted in their thigh. Immediately before, and 60 min after the cryotherapy exposure, the probe was withdrawn incrementally to ascertain the muscle temperature at 2 cm and 1 cm respectively below the subcutaneous fat layer. Before each treatment the probe was removed and the injection site covered with waterproof dressing.

### Skin Temperature

Skin temperature was assessed using a ThermoVision A40M Thermal Imaging camera (Flir Systems, Danderyd, Sweden) in accordance with the standard protocol of infrared imaging in medicine [27]. The camera, with the emissivity set at 0.97–0.98, was connected to a personal computer (Portege A100, Toshiba, Japan) with appropriate software (Thermacam Researcher Pro 2.8, Flir systems, Danderyd, Sweden). The camera was mounted on a tripod and the distance between the camera and the subject ranged from 3.7–4.2 m (depending on the height and the size of the individual). The validity and reliability of using noncontact, digital, infrared, thermal imaging (TI) cameras to measure skin surface temperature has previously been established [28] and we have recently reviewed the benefits of TI as a method of assessing skin temperature following cryotherapy [29]. To create a quadrilateral region of interest (ROI) around the thigh area an inert marker was placed 5 cm above the most superior aspect of the patella [29]. This marker created the inferior horizontal line of the quadrilateral, while the apex of the groin created the superior line during post process analysis [29]. The minimum, maximum and average skin temperature within this ROI on the subjects’ right thigh was manually recorded pre, immediately post and 10 min thereafter for 60 min after both treatments. The subjects wore shorts, stood for the duration of the testing period and were asked to remain in the anatomical position while images were being recorded.

### Core Temperature

To record core temperature, a rectal probe (MRV Adult Rectal Probe, Ellab A/S, Hvidovre, Denmark) was inserted by the volunteers 10–12 cm beyond the external anal sphincter. The thermistor was connected to a portable thermometer (DM852, Ellab A/S, Hvidovre, Denmark) and the rectal temperature was recorded manually using the same protocol as skin temperature. Subjects were acclimatised to the temperature of the room, wearing the clothing described above, for 40 min to obtain a stable baseline during skin and core temperature recording.

### Thermal Comfort and Thermal Sensation

Ratings of thermal sensation were recorded every 5 min throughout the duration of the study. Subjects were asked to rate their thermal sensation on a nine point standard scale [15] before and after both WBC and CWI. Subjects were asked ‘How are you feeling now?’ and then answered by pointing to a scale from −4 to 4. 4 = very hot, 3 = hot, 2 = warm, 1 = slightly warm, 0 = neutral, −1 = slightly cool, −2 = cool, −3 = cold, −4 = very cold). Thermal comfort [30] was also assessed immediately after exposures with a five-point scale (‘Do you find this,’ 0 = comfortable, 1 = slightly uncomfortable, 2 = uncomfortable, 3 = very uncomfortable, 4 = extremely uncomfortable). The subjects were instructed to relate their sensations at the time of reporting.

### Reliability

The test-retest reliability of the current methodology (using mean differences and 95% limits of agreement) was established using the value of the pre-WBC subtracted from the pre-CWI trials in the 20 subjects. The mean difference (SD) for muscle temperature between the two trials was −0.13 (1.21), 0.25 (0.79) and 0.02 (0.77) °C for 1, 2 and 3 cm subcutaneous respectively. Limits of agreement (mean difference ±1.96 x SD) for muscle temperature at a depth of 1, 2 and 3 cm ranged from –2.5 to 2.24, −1.31 to 1.81 and −1.48 to 1.52°C. For skin temperature the mean differences were −0.29 (0.43), −0.14 (0.49) and −0.4°C (0.6) for average, minimum and maximum temperature respectively. The limits of agreement for skin temperature were −1.12 to 0.54, −1.11 to 0.82 and −1.67 to 0.87°C respectively. Core temperature between trials had a mean difference of −0.1°C (0.22) with limits of agreement ranging from −0.53 to 0.33°C.

### Statistical Analysis

All data are presented as group means and SD. We performed a priori analysis where the final baseline (°C) recording of muscle, skin (minimum, maximum and average) and core temperature was compared to that of the post treatment temperature at 0, 10, 20, 30, 40, 50 and 60 min following both cryotherapy modalities. A two-way repeated measures ANOVA (treatment x time) was used to investigate changes in time with one between-subjects variable, treatment, with two levels (WBC and CWI) and one within-subject variable, time, with eight levels (baseline (recording immediately before treatment), 0, 10, 20, 30, 40, 50 and 60 min post) for muscle (3 cm subcutaneous), skin (minimum, maximum and average) and core temperature. In addition, to determine the relationship between treatment and muscle temperature depth, we ran a further repeated measures AVOVA (treatment x time x subcutaneous depth) with one between-subjects variable, treatment, with two levels (WBC and CWI) and two within-subject variable, time (pre, post) and subcutaneous depth (1, 2, 3 cm). The effect of time, treatment, and treatment by time interactions were tested in all analysis. When the main effect was significant, a Bonferroni adjusted post-hoc test was used to investigate within-group differences. To examine if baseline data, before CWI and WBC, were similar for muscle, skin or core temperature, a paired sample t-test was employed. All variables were tested for normality using the Shapiro-Wilk test. When the assumption of sphericity was violated, significance was adjusted using the Greenhouse-Geisser method. The current study had an 80% power to detect a difference of 1°C in muscle, 1°C in skin and 0.25°C in core temperature between conditions. Ratings of thermal comfort were analysed using the Wilcoxon signed-rank test. A Friedman test was used to detect differences across time for the nonparametric data obtained from the Likert-type measurement scale for thermal sensation. A follow-up analysis using the Wilcoxon signed-rank procedure, with a Bonferroni correction, was used to examine differences between baseline and data obtained during each follow-up. All statistical analyses were performed in SPSS (Statistical Package for the Social Sciences), version 19.0 (SPSS Inc, Chicago, IL) with the level of significance set at P<0.05.

## Results

### Muscle Temperature

**Figure 1:**
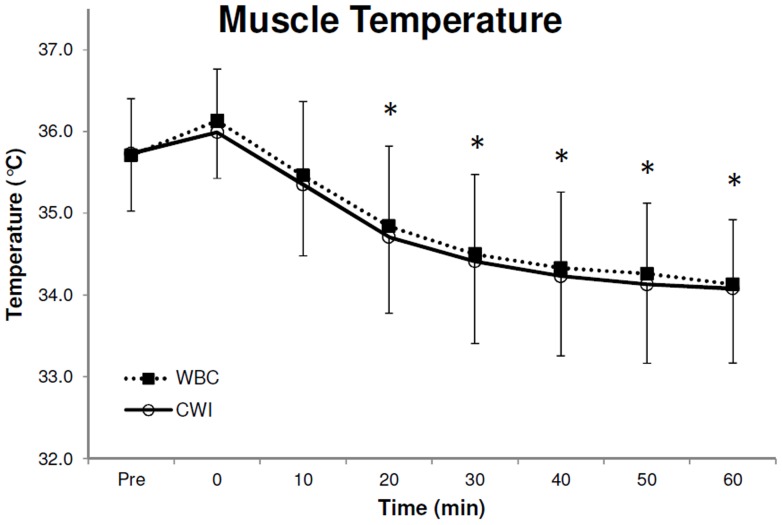
Muscle temperature (recorded 3 cm below the subcutaneous fat layer in the right Vastus lateralis), before (1a) and after (1b) both Cold Water Immersion (CWI) and Whole Body Cryotherapy (WBC). Values are means ± SD (N = 10). *Statistical significance (P<0.05) observed over time (between pre and post) conditions for both modalities.

**Figure 2:**
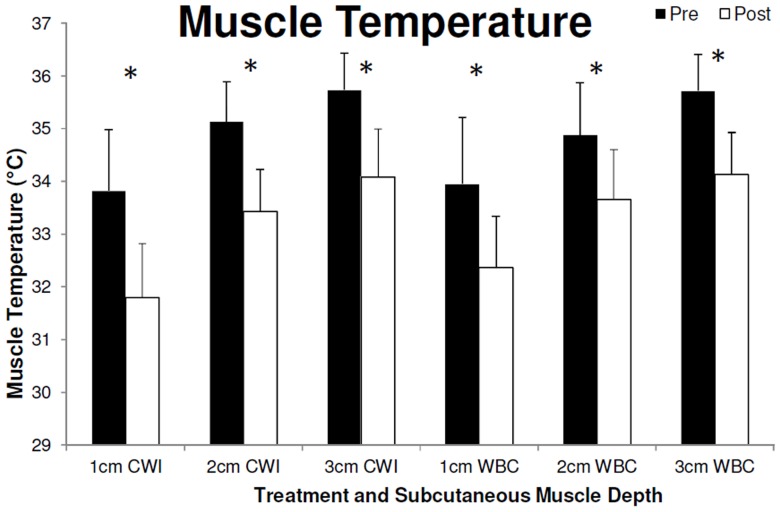
Muscle temperature (recorded 1, 2 and 3 cm below the subcutaneous fat layer in the right Vastus lateralis), after both Cold Water Immersion (CWI) and Whole Body Cryotherapy (WBC). Data recorded 1 hr after exposure. Values are means ± SD (N = 10). *Statistical significance (P<0.05) observed over time (between pre and post conditions) for both modalities.

**Figure 3:**
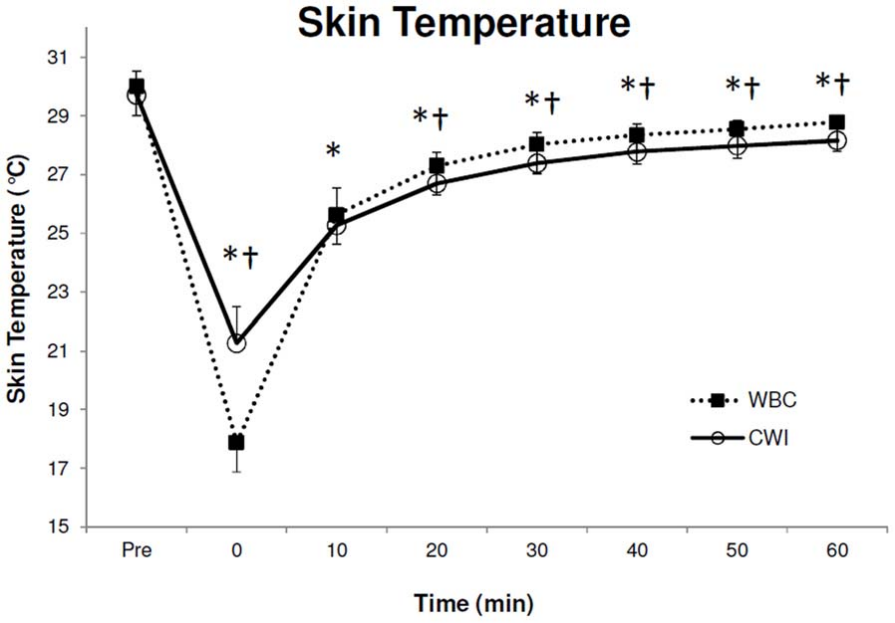
Average thigh skin temperature before (3a) and after (3b) both Cold Water Immersion (CWI) and Whole Body Cryotherapy (WBC). Values are means ± SD (N = 10). Statistical significance (P<0.05) observed over time* (between pre and post conditions) and between modalities^†^.

Baseline temperature in the vastus lateralis muscle before CWI and WBC were similar (P>0.05 [paired sample t-test]) at a probe depth of 1 cm (WBC, 34.0±0.7; CWI, 33.8±1.2°C), 2 cm (WBC, 34.9±1.0°C; CWI, 35.1±0.8°C) and 3 cm (WBC, 35.7±0.7°C; CWI, 35.7±0.7°C). A significant effect over time was observed in deep muscle temperature (3 cm subcutaneous; F_7,63_ = 70.2, P<0.001, 1−β = 1.0). Post-hoc tests displayed significantly lower temperatures (P<0.05) compared to baseline at 20, 30, 40, 50 and 60 min after both WBC and CWI, at a depth of 3 cm (Fig. 1). No between treatment differences were observed in deep muscle temperature (F_1,9_ = 0.1, P = 0.717, 1−β = 0.063). A significant difference between pre and post, CWI and WBC, was observed at a depth of 1, 2 and 3 cm (F_1,9_ = 120.1, P<0.001, 1−β = 1.0), however no between treatment differences were observed (F_1,9_ = 0.4, P = 0.560, 1−β = 0.084). Muscle temperature 60 min after treatment at the different depths was as follows; 1 cm (WBC, 32.4±1.0°C; CWI, 31.8±1.0°C; Fig. 2), 2 cm (WBC, 33.7±0.9°C; CWI, 33.4±0.8°C; Fig. 2) and 3 cm (WBC, 34.1±0.8°C; CWI, 34.1±0.9°C, Fig. 2). Despite observing a higher temperature pre and post treatment at a depth of 3 cm compared to 2 cm and 1 cm, and 2 cm compared to 1 cm (F_2,18_ = 129.8, P<0.001, 1−β = 1), no other significant differences (P>0.05) were observed in muscle temperature.

### Thigh Skin Temperature

Baseline thigh skin temperatures for each modality were similar before treatment (P>0.05 [paired sample t-test]; Table 1). Similar to muscle temperature there was a significant reduction in average (F_7,63_ = 1001.4, P<0.001, 1−β = 1.0; Fig. 3), minimum (F_7,63_ = 709.7, P<0.001, 1−β = 1.0; Table 1) and maximum (F_7,63_ = 141.8, P<0.001, 1−β = 1.0; Table 1, Fig. 3) skin temperature over time. Post-hoc analysis showed these differences occurred at 0, 10, 20, 30, 40, 50 and 60 min after both treatments compared to baseline (P<0.05) in average, minimum and maximum skin temperature. A significant treatment by time effect was also observed in average (F_7,63_ = 105.5, P<0.001, 1−β = 1.0), minimum (F_7,63_ = 52.4, P<0.001, 1−β = 1.0) and maximum (F_7,63_ = 7.2, P<0.001, 1−β = 1.0) skin temperature. Average, minimum and maximum skin temperature was significantly lower (P<0.05) immediately after WBC compared to CWI. However, average skin temperature from 20 to 60 min, minimum temperature 40 and 60 min and maximum skin temperature at 10, 30, 40, 50 and 60 min was significantly lower after CWI compared to WBC (P<0.05).

**Table 1:** Comparison of right thigh skin temperature change after exposure to Cold Water Immersion (CWI) and Whole Body Cryotherapy (WBC).

	WBC Pre (°C)	WBC Post (°C)	WBC ΔT (°C)	CWI Pre (°C)	CWI Post (°C)	CWI ΔT (°C)
Average	30.0±0.8	17.9±1.4	12.1±1.0*	29.7±0.8	21.3±1.2	8.4±0.7*
Min	28.9±0.8	15.7±1.5	13.2±1.4*	28.8±0.7	20.1±1.0	8.7±0.7*
Max	31.5±1.0	22.7±2.3	8.8±2.0*	31.1±1.2	23.9±2.7	7.2±1.9*

Values are means ± SD (N = 10).

* Statistical significance (P<0.05) between pre and post conditions. ΔT; temperature difference between pre and post treatment.

### Core Temperature

**Figure 4:**
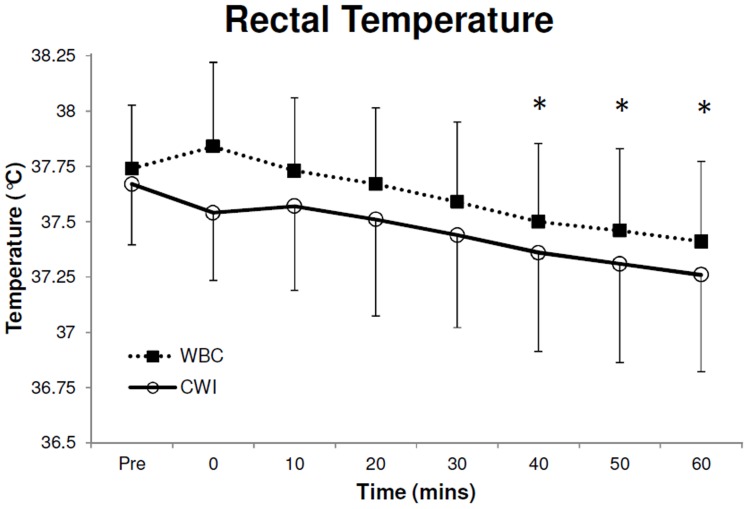
Rectal temperature before (4a) and after (4b) both Cold Water Immersion (CWI) and Whole Body Cryotherapy (WBC). Values are means ± SD (N = 10). *Statistical significance (P<0.05) observed over time (between pre and post treatment) for both treatment exposure.

Baseline rectal temperate before CWI and WBC were similar (WBC, 37.7±0.3°C; CWI, 37.7±0.3°C; P>0.05 [paired sample t-test]). A decline in rectal temperature was observed over time (F_7,63_ = 24.7, P<0.001, 1−β = 1; Fig. 4). Significant reductions (P<0.05) in rectal temperature occurred at 40, 50 and 60 min after treatment. However, there were no significant differences at any time between treatments (P>0.05). The greatest reduction from baseline was observed 60 min after WBC (0.3±0.2°C) and CWI (0.4±0.2°C).

### Thermal Comfort and Thermal Sensation

Subjects tended to find the WBC (2.7±1.4; indicating ‘uncomfortable’) exposure more uncomfortable (Z = −2.55, P = 0.011) than CWI (1.8±1.1; indicating ‘slightly uncomfortable’). Significant differences were observed in thermal sensation 0 min (WBC, −1.8±1.8; CWI, −1.4±1.3) and 5 (WBC, −0.7±1.3; CWI, −0.7±1.2) min post both modalities compared to their respective baseline sensation (WBC, 0.4±0.7; CWI, 0.4±0.5; P<0.05). During a post-hoc analysis using the Wilcoxon signed-rank tests no significance were found between CWI and WBC (P>0.05).

## Discussion

The present study used a randomised controlled crossover design to establish the effect of −110°C WBC, an increasingly popular method of cryotherapy in sports medicine, on muscle, skin and core temperature. To our knowledge this study is the first to (a) establish the effects of −110°C WBC on intramuscular temperature and (b) compare the muscle, skin and core temperature cooling potential of both WBC and a more established method of cryotherapy, CWI.

Several modalities of cryotherapy are currently employed by physiotherapists, sports physicians and physical therapists for athletic recovery, injury and rehabilitative purposes to reduce tissue temperature, nerve conduction velocity and to provide analgesia. As heat conductance is greater in water than in air [16] we hypothesized that WBC would not reduce muscle temperature to the same degree as CWI. This hypothesis has to be rejected as a similar reduction, in both deep and superficial muscle temperature, was observed in a population of healthy active males after both treatments. Significant differences compared to baseline were observed for both treatments at 20, 30, 40, 50 and 60 min post treatment (Fig. 1). Deep muscle temperature (3 cm) continued to decline up to 60 min after both treatments. Interestingly, there were no significant differences between the WBC and the CWI at any time and both modalities display very similar reductions in muscle temperature following treatment (Fig. 1). Similarly, there was also a significant reduction in superficial muscle temperature (1 cm and 2 cm subcutaneous) after treatment but no differences between treatments were observed (Fig. 2).

Although researchers have not reached consensus regarding ideal reductions in muscle or skin temperature [31] it has been suggested that, in the absence of definitive data, better treatment outcomes (e.g. analgesia following acute injury) may result from greater and faster cooling [26], [32]. Reductions in muscle temperature following various modalities of cryotherapy including cold packs [5], ice packs [33], CWI [11], [34] and deep freeze cooling gel [35] are widely reported within the literature. Although previous studies have also shown the WBC is effective in reducing tympanic [1] and skin temperature [14], [15] it has, until now, yet to be fully elucidated whether or not WBC is effective in reducing superficial or deep muscle temperature. We have previously highlighted the need to establish the potential of WBC to reduce muscle temperature [1] and these data will help inform sport physician, physiotherapists, coaches and athletes alike.

The CWI methodology employed in the current study was similar to that of Gregson and colleagues [11] who assessed muscle temperature (1, 2, 3 cm below the subcutaneous fat layer in the vastus lateralis) after immersion in 8°C water for 10 min. Despite employing a similar protocol in terms of water temperature, these authors reported a greater reduction (3 cm subcutaneous) in muscle temperature of ∼0.5°C than the current study, 30 min after immersion. The duration of immersion employed (6 min longer than the current study) and the fact that the legs were not dried in the study by Gregson [11] may explain this discrepancy. The reductions in muscle temperature observed after a 4 min exposure to WBC and CWI do not compare well to that of other cryotherapy modalities. Muscle temperature in the calf (3 cm subcutaneous) has been shown to be reduced by almost 8°C after a 20-min crushed-ice pack application [36]. Furthermore, a 30-min application of Wet-Ice has been shown to reduce quadriceps muscle temperature by 5.62°C and 8.44°C, at 1 cm and 2 cm subcutaneously respectively [26].

Interestingly, greater absolute reductions in more superficial muscle temperature, compared to the deeper muscle, were not observed following cryotherapy in the current study (Fig. 2). Similar, data were provided by Enwemeka and colleagues who found the reduction in superficial muscle temperature (1 cm) to peak 20 min after cold pack application and rose rapidly thereafter [5]. However, as the temperature at a depth of 3 cm was recorded continuously in the current study, muscle temperature at a depth of 1 and 2 cm subcutaneous was only recorded 60 min after treatment (when the probe was removed incrementally). It is likely that the more superficial muscle temperature, especially 1 cm subcutaneous, had a similar temperature decline and recovery to that of the skin temperate. This may be attributed to a progressive reversal of the temperature gradients of the superficial and deep tissues, with the deeper tissues losing heat (cooling) simultaneously as the superficial tissues were rewarming to attain their initial baseline temperatures [5].

Although significant reductions in tissue temperature are believed to be required to reduce inflammation [31] and create and analgesic response [29], it should be noted that reductions in tissue temperature may be delirious to functional performance [31] and proprioceptive acuity [1], [24], [37]. This is troublesome if exercise is resumed following cryotherapy, a protocol commonly employed before exercise in hot and humid environments. A thorough review of the effects of other modalities of cryotherapy on tissue temperature is provided elsewhere [13], [29].

A significant reduction in average (Fig. 3 and Table 1), minimum (Table 1) and maximum (Table 1) skin temperature was observed after both treatments. Despite the similar reductions in skin temperature after both treatment modalities, we observed evidence of a greater reduction in thigh temperature immediately after WBC (Fig. 3). However, both modalities display different recovery patterns and average skin temperature after CWI was significantly lower than WBC at 20, 30, 40, 50 and 60 min after treatment. In order to record accurate TI data, moisture from the CWI would affect the analysis of skin temperature, the subjects in the current study were asked to towel dry after immersion and this friction may have helped to slightly increase skin temperature. As towel drying is an artefact of CWI, we considered this an integral component of the treatment.

The present findings of a skin temperature reduction of 12.1±1.0°C in the thigh immediately after WBC exposure is similar to that reported elsewhere after WBC [14], [15]. Furthermore, using a similar CWI protocol Gregson and colleagues [11] have also reported a comparable reduction of ∼10°C (8.4±0.7°C in the current study) in thigh skin temperature after a 5 min treatment. Although a skin temperature reduction of ∼2°C less than these results were found after CWI in the current study it is possible that the extra min of immersion employed by Gregson [11] explains the slight discrepancy. Similar to muscle temperature, these findings do not compare favourably to other modalities of locally applied cryotherapy such as ice bag or wet ice application. Other investigators have reported skin temperature reductions of between 21–25°C following the local application of various types of cryotherapy for 20–30 min [32]. Based on the information reported by Bleakley and Hopkins [13] it can be deduced that, although a skin temperature reduction of within the 5–15°C range was reached after both CWI and WBC in the thigh, analgesia was not achieved as the skin temperature remained above the critical 12°C mark. Similarly, as a skin temperature of 12.5–13.5°C is required to observe a 10% decrease in nerve conduction velocity [38], although not measured as part of the current methodology, it can be assumed that a reduction in nerve conduction velocity of less than 10% would have been observed following both treatments. Although skin temperature following WBC has previously been reported, to our knowledge, this is the first study that aimed to compare and contrast skin temperature reduction, and indeed recovery, following WBC and CWI in a randomised crossover design. Consequently, these findings may be useful to clinicians, physiotherapists, athletic trainers and other researchers, who intend to utilise cryotherapy to reduce skin temperature to a safe degree.

A reduction in rectal temperature was observed 40, 50 and 60 min after both treatments compared to baseline (Fig. 4), but no significance was observed between treatments. Our results are similar to the reductions reported by others after CWI’s [11], but data on the effects of WBC on core temperature are scant. Although, we have previously shown that WBC reduces tympanic temperature by 0.3°C [1], it has previously been reported that WBC exposure does not cause a change in rectal temperature in an older population (48±7.9 years) [15]. However, Westerlund and colleagues [15] utilised an exposure duration of 2 min shorter than the current study and only recorded data for 30 min after exposure despite a steady decline similar to the current study.

The thermodynamics of cryotherapy, or cooling, works on the principle that heat is transferred unidirectional from high heat to low heat [16] and tissue temperature loses heat to the external cooling modality. Our results fit well with this concept as we see muscle temperature fall and the skin temperature increase after treatment. This transfer of heat from one body to another depends on several factors including the relative masses of the bodies, the size of the contact area, the difference in starting temperatures, the heat capacity of each material and the re-warming of the tissue from its own metabolic activity and perfusion [32]. Considering the methodology of the two cooling modalities employed in the current study (WBC and CWI) the duration was the same (4 min), the masses of the bodies were the same during both treatments (randomized crossover), the contact area was similar (whole body versus heat out immersion) and the baseline temperature of the skin was similar. The major discrepancies between the modalities in the current study were the temperature difference of the two treatments (∼118°C), the hydrostatic pressure of water and the difference in the ability of air and water to conduct heat.

The temperature of the muscle, skin and core did not return to baseline levels 60 min following treatment and this agrees with the finding of others [29]. Furthermore, rectal temperature continued to decline up to 60 min after treatment. It has previously been reported that it may take as long as 4 hours for tissue temperature to return to baseline following cooling and both Merrick et al. [32] and Enwemeka et al. [5] have highlighted the need for continued monitoring of tissue temperature following the removal of the cooling agent. Therefore, an extended period of temperature recording (60 min after both treatments) was utilised in the current study. The results of this study are limited to a homogenous group of active healthy male subjects, between the ages of 18 to 35, as the depth of subcutaneous fat is a significant factor in the magnitude and rate of intramuscular cooling. The insulation effects of adipose tissue during cryotherapy treatment and re-warming after treatment has previously been established [36]. The mid-thigh skinfold thickness of this group was 10.5±5.9 mm and these results are similar to that of other studies using physically active male volunteers [39]. Future research studying the effects of WBC is therefore warranted on females, who tend to have a different percentage and distribution of body fat.

One limitation of the present study was that methodological constraints did not allow the assessment of muscle, skin and rectal temperature on the same subjects at the same time. However, we used a homogeneous population and the treatment protocols conditions remained constant throughout. As the equipment used in the current study was not functional in temperatures below −100°C, skin and rectal temperatures were not recorded during WBC or CWI. Furthermore, in an attempt to reduce the risk of infection, muscle temperature was not recorded during the treatments. As superficial muscle temperature was only recorded 60 min after treatment it is possible that larger reductions in the more superficial muscle temperature occurred before this time and future research is required to clarify this. Finally, we only assessed muscle, skin and core temperature after one treatment of CWI and WBC. As both these treatments, especially WBC, are often repeated several times on the one day future research is required to assess the dose response of both treatment.

### Conclusions

The present study demonstrates that a single WBC exposure (−110°C for 4 min) decreases muscle and core temperature to a similar level of those experienced after CWI (8°C for 4 min). Although both treatments significantly reduced skin temperature from baseline, WBC elicited a greater decrease compared to CWI. It is likely that a temperature deficit of 118°C experienced during WBC exposure compensated for the reduced thermal conductivity of air compared to water. These data may provide information to clinicians and researchers alike who utilise WBC and CWI protocols in a clinical or sporting setting. As such, further investigation is warranted to explore the effects of a reduction in tissue temperature following WBC and CWI in athletic recovery and acute injury.
